# Proteomics Analysis of Three Different Strains of *Mycobacterium tuberculosis* under *In vitro* Hypoxia and Evaluation of Hypoxia Associated Antigen’s Specific Memory T Cells in Healthy Household Contacts

**DOI:** 10.3389/fmicb.2016.01275

**Published:** 2016-09-09

**Authors:** Santhi Devasundaram, Akilandeswari Gopalan, Sulochana D. Das, Alamelu Raja

**Affiliations:** Department of Immunology, National Institute for Research in Tuberculosis (ICMR)Chennai, India

**Keywords:** *M. tuberculosis*, hypoxia, prevalent clinical strains, two-dimensional electrophoresis, mass spectrometry, multicolor flow cytometry

## Abstract

*In vitro* mimicking conditions are thought to reflect the environment experienced by *Mycobacterium tuberculosis* inside the host granuloma. The majority of *in vitro* dormancy experimental models use laboratory-adapted strains H37Rv or Erdman instead of prevalent clinical strains involved during disease outbreaks. Thus, we included the most prevalent clinical strains (S7 and S10) of *M. tuberculosis* from south India in addition to H37Rv for our *in vitro* oxygen depletion (hypoxia) experimental model. Cytosolic proteins were prepared from hypoxic cultures, resolved by two-dimensional electrophoresis and protein spots were characterized by mass spectrometry. In total, 49 spots were characterized as over-expressed or newly emergent between the three strains. Two antigens (ESAT-6, Lpd) out of the 49 characterized spots were readily available in recombinant form in our lab. Hence, these two genes were overexpressed, purified and used for *in vitro* stimulation of whole blood collected from healthy household contacts (HHC) and active pulmonary tuberculosis patients (PTB). Multicolor flow cytometry analysis showed high levels of antigen specific CD4^+^ central memory T cells in the circulation of HHC compared to PTB (*p* < 0.005 for ESAT-6 and *p* < 0.0005 for Lpd). This shows proteins that are predicted to be up regulated during *in vitro* hypoxia in most prevalent clinical strains would indicate possible potential immunogens. *In vitro* hypoxia experiments with most prevalent clinical strains would also elucidate the probable true representative antigens involved in adaptive mechanisms.

## Introduction

The success of *Mycobacterium tuberculosis* lies in its ability to persist within humans for long periods without causing any disease symptoms, known as latent tuberculosis infection (LTBI). About two billion people are estimated to have latent infections that could reactivate into TB disease (Kasprowicz et al., 2011). Because of the huge reservoir of latently infected individuals, diagnosis, and treatment of latent TB infections have obtained increasing importance as public health measures to control TB. In-depth knowledge about the biology of dormant *M. tuberculosis* is important to develop new therapeutic tools for latent TB (Barry et al., 2009). Several lines of evidence link latent tuberculosis and inhibition of *M. tuberculosis* growth with hypoxic conditions. Depletion of oxygen prevents aerobic respiration by the obligate aerobe within the host (Fang et al., 2012). In order to persist within the host, *M. tuberculosis* possess the ability to adapt to the hypoxic environment which is considered a crucial part of the adaptation mechanism (Muttucumaru et al., 2004).

To better understand this state of dormancy, numerous *in vitro* experiments have been developed to mimic, at least in part, the host’s intracellular environment experienced by *M. tuberculosis*. *In vitro* mimicking conditions include oxygen deprivation (hypoxia), low pH, and nutrient starvation which either inhibits or slows down bacterial growth (non-replicating stage). Among these, hypoxia is extensively studied and considered a potential factor for transformation into the non-replicating dormant form of *M. tuberculosis* (Rustad et al., 2009; Fang et al., 2012). The most frequently used experimental model for hypoxia-induced *M. tuberculosis* dormancy is the defined headspace model of non-replicating persistence (NRP; Wayne and Hayes, 1996) and is adapted for the present study.

To date, many *in vitro* hypoxia experimental models use common laboratory mycobacterial strains like H37Rv and Erdmann (Sherman et al., 2001; Voskuil et al., 2004). However, laboratory strains might not completely represent the virulence of naturally occurring clinical strains involved in disease outbreaks. A few hypoxia reports (Boon et al., 2001; Starck et al., 2004) used prevalent clinical strains but these strains were not from TB endemic areas. The unique feature of the present report is that we have used two prevalent clinical strains (S7 and S10) from a TB endemic region like India and evaluated their adaptation mechanisms, in terms of protein expression, under *in vitro* hypoxia.

The strains S7 and S10 were first reported by Das et al. (1995) from a restriction fragment length polymorphism (RFLP) study which showed that most (38–40%) of the clinical isolates of *M. tuberculosis*, taken from the *Bacillus* Calmette–Guerin (BCG) trial area of Tiruvallur district, south India, harbored a single copy of *IS6110* in their genome. Among the other strains studied by Rajavelu and Das (2005), S7, and S10 drew our attention due to their distinct immune responses (S7 induced Th-2 response while Th-1 response was induced by strain S10) despite having a single copy of *IS6110* at the same locus in the genome.

Genes/proteins that are over-expressed during *in vitro* stress are likely to be crucial for intracellular survival of *M. tuberculosis* and are potential targets for anti-TB drug and vaccine development (Betts, 2002; Andersen, 2007; Hingley-Wilson et al., 2010).

We hypothesized that proteins identified as up regulated especially in clinical isolates, during *in vitro* hypoxia, would be better potential vaccine candidates than proteins predicted from laboratory adapted strains.

To analyze hypoxia associated proteins, we compared protein expression profiles of each strain (H37Rv, S7, and S10) during well aerated growth conditions (aerobic) and oxygen depleted growth conditions (anaerobic/hypoxic).

Proteins spots, expressed under hypoxia were characterized by mass spectrometry. Two antigens were selected for *in vitro* recombinant antigen preparation to test our hypothesis of using clinical strains to obtain the most promising potential antigens. These two antigens were used to stimulate samples of whole blood collected from latently infected healthy household contacts (HHCs) and active TB individuals [pulmonary TB (PTB)]. The LTBI population is presumed to be protected against active TB disease and antigens that are preferentially detected by LTBI can be considered as novel vaccine targets (Andersen, 2007; Govender et al., 2010).

## Materials and Methods

### Mycobacterial Strains

The laboratory strain H37Rv (ATCC 27294) of *M. tuberculosis* was obtained from Colorado State University (CSU), Fort Collins, CO, USA. H37Rv was originally derived from H37, a clinical isolate isolated from a pulmonary tuberculosis patient in 1905 (Steenken et al., 1934). The clinical isolates S7 and S10 of *M. tuberculosis* were first isolated from the BGC trial area of the Tiruvallur District, Tamil Nadu, India during the Model Dots trial (Das et al., 1995). These isolates are maintained as glycerol stocks and can be obtained through proper request to National Institute for Research in Tuberculosis, Chennai, India.

### *In vitro* Culture Method

Three mycobacterial strains (H37Rv, S7, and S10) were grown in Middlebrook 7H9 media (MB7H9) containing 2% glycerol (v/v), 10% albumin-dextrose-catalase (ADC), and 0.05% Tween 80 (v/v) at 37°C 200 rpm to obtain aerobic cultures.

Wayne’s *in vitro* oxygen depletion method was followed to generate hypoxic(anaerobic) cultures (Wayne and Hayes, 1996). All three strains (H37Rv, S7, and S10) were inoculated into screw capped test tubes (20 mm × 125 mm, with a total fluid capacity of 25.5 mL) pre-filled with supplemented MB7H9. Test tubes were initially filled with 17 ml of MB7H9 broth leaving 8.5 ml head space to give a head to air space ratio of 0.5. After inoculation, these tubes were incubated at 37°C. Sterile 8-mm Teflon-coated magnetic stirring bars were used in hypoxic cultures to stir gently at 120 rpm. This stirring maintains the uniform dispersion and the rate of O_2_ depletion was under control.

The O_2_ depletion was monitored by reduction and decolorization of the methylene blue indicator. A final concentration of 1.5 μg mL^-1^ of sterile solution of methylene blue (Sigma-Aldrich, St. Louis, MO, USA) was added into the hypoxia cultures during inoculation. In *M. tuberculosis in vitro* cultures methylene blue decolorization starts when the dissolved oxygen concentration is declined below 3% (Leistikow et al., 2010). Hence, complete decolorization of methylene blue was taken to indicate oxygen depletion.

The culture tube containing supplemented MB7H9, methylene blue and no bacterial inoculum was set-up as a “blank.” Growth was measured at OD_600nm_ (optical density) in both aerobic and anaerobic cultures. Triplicate cultures of both aerobic and anaerobic were set up for each strain.

### Cytosolic Proteins Preparation

Triplicate aerobic and anaerobic cultures of H37Rv, S7, and S10 were harvested by centrifugation at 4000 × *g* for 15 min at 4°C. The pellets, from triplicate cultures, were washed twice with 40 mM Tris-buffer, centrifuged (4000 × *g*, 15 min, 4°C), and the supernatant was discarded. The pellets were resuspended in lysis buffer containing 20 mM Tris-HCl, 100 mM dithiothreitol (DTT), 1 mM PMSF (phenylmethylsulfonyl fluoride), complete protease inhibitor cocktail (Sigma-Aldrich, St. Louis, MO, USA), and 10 mg/mL lysozyme in ice. The cell membrane was then disturbed by ultra sonication (amplitude 40%) and homogenate was collected after high speed centrifugation (18000 × *g* for 25 min). Likewise, three cytosolic protein fractions for H37Rv, S7, and S10 were available and were separated by 2DE (two-dimensional electrophoresis) experiments.

### Two-Dimensional Electrophoresis (2DE)

Four hundred (400 μg) micrograms of cytosolic fraction proteins from H37Rv, S7, and S10, from both aerobic and anaerobic cultures, were taken and impurities were removed by a 2D cleanup kit (Bio-Rad Laboratories, Hercules, CA, USA). Protein concentration was then estimated by BCA Protein assay – Reducing agent compatibility kit (Thermo Fisher Scientific Inc, Waltham, MA, USA).

For first dimension isoelectric focusing (IEF), protein samples were solubilized in rehydration buffer (8 M urea, CHAPS 2%, ampholytes 3–7 and 4–6 and 15–100 mM DTT and separated by 17 cm immobilized pH gradient (IPG) strips (Bio-Rad Laboratories, USA) of pH range 4–7. Each IPG strip was rehydrated with 300 μl rehydration buffer containing 200 μg protein samples. IEF was performed at 20°C in an IEF cell as per manufacturer’s instructions (Bio-Rad Laboratories, Hercules, CA, USA) by following electrical conditions; 150 V for 15 min, end voltage was 10,000 V and volt hours were 40–60,000. The electrical limit was set at 50 μA per strips. After IEF strips were first equilibrated with equilibration buffer 1 (2% DTT, 2% SDS, and 6 M urea) for 10 min and followed by equilibration buffer 2 (2.5% iodoacetamide, 2% SDS, and 6 M urea) for 10 min. Each of the IPG strips was loaded on a vertical SDS-PAGE gel (12%; Second dimension) and sealed with 1% low melting agarose dissolved in SDS running buffer. SDS-PAGE was performed under reducing conditions at constant voltage (150 V). Then, gels were developed by Coomassie brilliant blue (CBB) R 250 (Bio-Rad Laboratories, Hercules, CA, USA) to visualize proteins and images of gels were acquired by Chemidoc XRS gel documentation system (Bio-Rad Laboratories, Hercules, CA, USA). Image analysis was performed using the PDQuest software (version 7.0.0; Bio-Rad Laboratories, Hercules, CA, USA) by stepwise spot detection and spot matching. Protein samples from all three strains were loaded in equal concentrations and 2-DE (two-dimensional electrophoresis) experiments were done for all three lysates prepared per strain. Both aerobic and anaerobic lysates were loaded simultaneously for IEF and SDS-PAGE to maintain the same running conditions. Uniform staining and destaining was followed for all gels (aerobic and anaerobic).

To identify protein expression differences between the aerobic and anaerobic cultures of all three *M. tuberculosis* strains, cytosolic proteins were separated by 2-DE and protein profiles were compared by PDQuest software. A threshold value of 1.5-fold difference was assigned to identify differentially expressed protein spots between the aerobic and anaerobic cultures of each strain. Student’s *t*-test with confidential hit 0.05 reliability score was performed to evaluate the significance of each differentially expressed protein spot. Differentially expressed protein spots that were identified consistently in two out of three cytosolic fraction’s 2-DE experiments were only included for further characterization.

Two types of criteria were used to identify the possible hypoxia associated proteins from these strains. First, protein spots whose intensity was higher in anaerobic gel (hypoxic) compared to its aerobic counterpart gel (aerobic) were selected and designated as “over-expressed” proteins during hypoxia. These “over-expressed” proteins are expressed at basal levels during aerobic growth and increase expression in a hypoxic environment. Secondly, protein spots which were completely absent (no basal level expression) during aerobic growth, but expressed only during anaerobic growth were selected and designated as “newly expressed” protein spots. These newly expressed spots were unique to anaerobic gel (hypoxia) and no corresponding spots were seen in the aerobic counterpart gel (aerobic).

### In-gel Digestion with Trypsin

Protein spots of interest were excised from gels and digested with modified trypsin (Roche Molecular Biochemicals, Indianapolis, IN, USA). The gel plugs were washed several times with 100 mM ammonium bicarbonate (NH_4_HCO_3_) in 50% acetonitrile, the gel pieces were subjected to a reduction step using 10 mM DTT in 100 mM NH_4_HCO_3_ buffer (45 min at 56°C). Alkylation was performed with a solution of 55 mM iodoacetamide in 100 mM NH_4_HCO_3_ (30 min at room temperature in the dark) followed by in-gel digestion with 20 μl of trypsin (10 ng/μl) in 50 mM NH_4_HCO_3_ (overnight at 37°C). Subsequently, the peptides were extracted in NH_4_HCO_3_ buffer with 5% formic acid. Samples were vacuum-dried and reconstituted in 25 μl of sample preparation solution (98% water, 2% acetonitrile, and 0.5% formic acid).

### Mass Spectrometric Analysis

The protein digest spectrum was acquired on a Q-STAR Elite (QTOF) mass spectrometer equipped with Applied Biosystems (Waltham, MA, USA) Nano Spray II ion source. The solution containing peptides was injected into Nano-LC through an autosampler system and then eluted with a gradient of water and acetonitrile. The flow rate of nano-reverse phase column (Michrom C18 5 μ 300 Å) was 400 nL/min for 1 h. This nano-reverse phase column is connected to the Nano Spray ESI- QTOF system (Qstar Elite, Applied Biosystems, Waltham, MA, USA). Eluted peptides from the column were ionized using ESI source with ion spray voltage 2250 V and temperature 120°C. Ionized peptides were analyzed by one full MS scan and four consecutive product ion scans of the four most intense peaks, using rolling collision energy. Ion fragmentation included a selection of ions in m/z range: >400 and <1600, of charge state of +2 to +5, exclusion of former target ions for 30 s, accumulation time of 1 s for a full scan and 2 s for MS/MS.

The resultant MS/MS data was searched against the NCBI non-redundant database^1^ option in the Protein Pilot 5.0 software (AB Sciex, Haryana, India) for the identification of proteins. During the analysis, in the search, parameter scope was allowed to include modification of cysteine by iodoacetamide and biological modifications programmed in algorithm were allowed. Mass tolerance for precursor ion and fragment ions were set to 100 ppm and 0.2 Da, respectively. The number of missed cleavages permitted was two. Differentially expressed proteins during hypoxia, in H37Rv, S7, and S10 were categorized according to function based on “TubercuList” database^2^.

### Study Subjects and Antigens Used for *In vitro* Whole Blood Culture

This study was approved by the Institutional Ethics Committee of National Institute for Research in Tuberculosis (NIRT) and informed consent was obtained from all study participants. We included 20 individuals (10 HHC and 10 PTB) patients who visited in Government Thiruvateeswarar Hospital of Thoracic Medicine, Otteri, Chennai. HHC participants, generally parents, spouses, and children, were selected if only they shared the living quarters for a minimum of 3 months with 10 h per day of close contact with sputum positive, active TB patients (index TB case) who were naive for anti-tubercular therapy. Their infection state was confirmed by positive QuantiFERON-TB Gold in Tube (QFT-IT; Cellestis, a company of Qiagen GmBH) results. All HHC were negative for acid fast bacilli sputum smear microscopy and had a negative chest x-ray indicating no active TB disease symptoms.

Pulmonary TB patients were recruited by positive sputum smear microscopy. Three sputum samples were collected at various days from all the active TB patients for smear and cultures. All PTB patients were positive for culture and were excluded if they had symptoms of immunosuppressive disease like diabetes, HIV, or other co-infections. All PTB patients were naive for anti-tuberculosis treatment.

Five ml of blood was collected and diluted 1:1 with RPMI1640 (Sigma-Aldrich, St. Louis, MO, USA) medium with penicillin/streptomycin (100 U/100 mg/mL), L-glutamine (2 mM), and HEPES (10 mM) and distributed into tissue culture plates. The cultures were then stimulated, at the final concentration of 5 μg/ml as determined earlier (Kumar et al., 2010), with *M. tuberculosis* ESAT-6 (E6), which was received from CSU, USA and Lpd (Rv0462) obtained by *in vitro* cloning and overexpression (Devasundaram et al., 2014) along with no antigen control (unstimulated). Phytohemeagglutinin (PHA) was used as mitogen control to show the proliferative capacity of lymphocytes from donors at a concentration of 1 μg/ml. Plates were incubated for 16 h with the prior addition of Brefeldin A (10 mg/mL) at fourth h. After incubation, cells were harvested with PBS and RBC were lysed with BD FACS lysing solution (Becton Dickinson, San Jose, CA, USA) as prescribed by the manufacturer. The cells were then stained for memory T cell markers.

### Surface Staining of Memory T Cells and Data Analysis

A single cell suspension of antigen stimulated cells was prepared with PBS and stained with antibodies against CD3 and CD4 conjugated to PerCP 5.5, APC-Cy7 (BD Biosciences, USA), respectively. Cells were then stained with PE-CD62L, a reliable surface marker to distinguish central and effector memory T cell subtypes. In addition, APC-CD45RA was used to differentiate naive cells from central and memory T cells. All antibodies were used at a final concentration of 5 μl/1 million cells and were incubated for 20–30 min in the dark followed by washing with PBS. Stained cells were immediately analyzed on a FACSCanto II flow cytometer with FACSDiva software, version 6 (Becton Dickinson and Company, Cockeysville, MD, USA). A total of 100, 000 lymphocyte events were recorded via forward and side scatter and data were analyzed in Flow Jo software (TreeStar). All data were depicted as the percentage of CD4^+^ T cells expressing memory surface markers.

CD3^+^ CD4^+^ T-cells were gated for expression of CD45RA and CD62L and defined as central memory (CD45RA^-^ CD62L^+^), effector memory (CD45RA^-^ CD62L^-^), naive (CD45RA^+^ CD62L^+^), and CD45RA^+^ effectors (T_EMRA_; CD45RA^+^ CD62L^-^).

For all antibodies utilized, fluorescence-minus-one (FMO) controls were used to define positive and negative boundaries. Compensation was calculated with signals from fluorochrome monoclonal antibodies linked to CompBeads (purchased from BD Biosciences, USA). The Mann–Whitney *U* test was performed using GraphPad Prism software (version 5.0; GraphPad Prism) with *p* < 0.05 considered to be statistically significant.

## Results

### Growth Patterns in Aerobic and Anaerobic Cultures of H37Rv, S7, and S10

**FIGURE 1:**
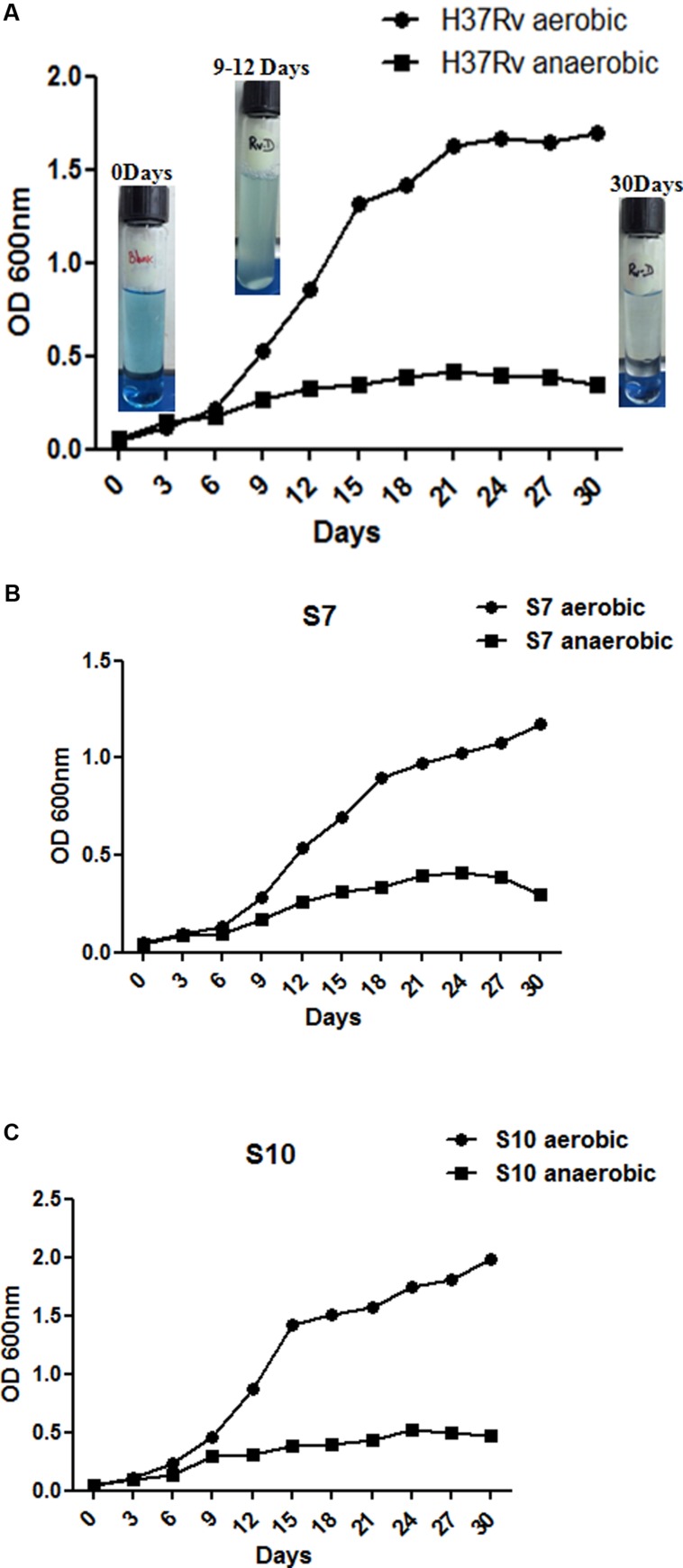
**Growth curves for *Mycobacterium tuberculosis* H37Rv aerobic and anaerobic cultures over the time course studied. (A–C)** Show growth patterns of H37Rv, S7, and S10, in duplicates, under aerated and anaerobic culture conditions, respectively. Aerated cultures of all three strains were obtained by growing at 37°C at 200 rpm with loose cap tubes. Dormant cultures (denoted as “D” to the suffix of each strain name) were obtained by growing all three strains for 30 days at 120 rpm in 20- by 125 mm screw-cap tubes containing MB7H9 broth. Cultures were stirred with 8 mm magnetic bars. Exemplary photos of H37Rv anaerobic cultures are given inside the graph. Mean values with standard error from duplicate cultures, at optical density (OD), 600 nm are shown.

At intervals, tubes (both aerobic and anaerobic) were removed for growth measurements at OD _600nm_ and methylene blue decolorization was also monitored. Similar patterns of growth curves were observed in both aerobic and anaerobic *in vitro* cultures. Complete decolorization of methylene blue indicated oxygen depletion in the anaerobic cultures (exemplary images of **Figure 1A**). Initial growth patterns were similar up to 5 days for both aerobic and anaerobic cultures in all three strains (**Figures 1A–C**) but growth declined from day 9 in hypoxic culture tubes. Decolorization of methylene blue was also similar in three strains under hypoxia. The “blank tube” remained the same color till the end of incubation as no bacterium was inoculated, as observed in our earlier experiment (Devasundaram et al., 2015).

### Protein Expression during Hypoxia in H37Rv, S7, and S10

Aerobic and anaerobic cultures were harvested at 30 days and complete methylene blue decolorization in anaerobic cultures was observed between 25 and 30 days.

**FIGURE 2:**
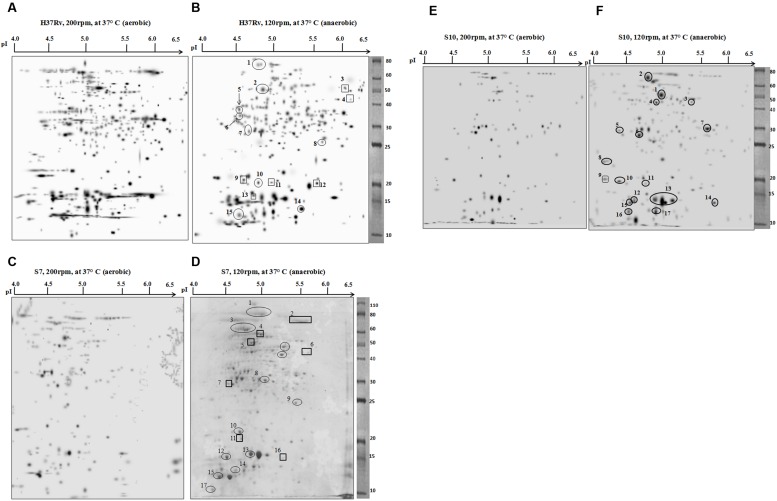
**Two-dimensional electrophoresis (2DE) gels of *M. tuberculosis* strain H37Rv, clinical isolates S7 and S10.** Representative 2DE gel pictures, produced by PDQuest software, from the cytosolic protein fractions of each strain are given. The panels **(A,C,E)** show 2DE separated protein fractions of aerobic cultures of H37Rv, S7, and S10, respectively. Likewise, the panels **(B,D,F)** Represent 2DE separated protein fractions of anaerobic cultures of H37Rv, S7, and S10, respectively. Spot details are given in the corresponding tables. Spots that are encircled indicate overexpressed protein spots during hypoxia and spots that are boxed are newly appeared protein spots during hypoxia condition.

Two-dimensional electrophoresis gels were stained with CBB R 250 as prescribed (Sharma et al., 2010; Kumar B. et al., 2013). Spots with a consistent increase in intensity (over-expressed spots) and spots that emerged only during hypoxia (newly appeared) were selected and identified by mass spectrometry. The majority of cytosolic proteins from *M. tuberculosis* H37Rv, the clinical strains S7 and S10 were focused in the acidic pH range of 4–6.5. 2-DE gels of cytosolic proteins under aerobic and anaerobic growth conditions are shown in **Figure 2**.

By comparing the protein spots between aerobic and anaerobic cultures of H37Rv, a total of 15 spots either over-expressed (encircled) or newly appeared (boxed) during hypoxia were identified, designated as RvD (H37Rv dormant) and numbered sequentially (**Figures 2A,B**). The isoelectric point (pI) and molecular weight (Mr) of the proteins were identified on the gel against each spot position. Details of the open reading frame (ORF) number, predicted gene product, number of matched peptides, and the percentage sequence coverage obtained for each protein spots are given in **Table 1**. A few differentially expressed protein spots, identified by PDQuest from hypoxic conditions, were not characterized by mass spectrometry. This was either due to low concentration or because a confident peptide match was not obtained during the peptide search in Protein pilot software. The majority of spots in the gel were of single protein identity while a few proteins were found to exist as multiple spots (spot RvD1 and RvD2 in **Figure 2B**).

**Table 1:** Overexpressed and newly appearing proteins identified by mass spectrometry from *Mycobacterium tuberculosis* laboratory strain H37Rv under hypoxia compared to aerated cultures.

Spot no	Rv no	Protein Name	Predicted M.Wt (kDa)	Predicted pI	Coverage (%)	Peptide matched
RvD1	Rv0440	GroEl2	65	4.8	7.60%	TDDVAGDGTTTATVLAQALVR, GLNALADAVK
RvD2	Rv1240	Probable malate dehydrogenase (MDH)	48	4.9	16.7%	NAAEVVNDQAWIEDEFIPTVAK, SDLLEANGAIFTAQGK
RvD3	Rv0462	Dihydrolipoamide dehydrogenase (LpdC)	50	5.8	35%	DAKAFGISGEVTFDYGIAYDR, VAGVHFLMK, LVPGTSLSANVVTYEEQILSR, ATFCQPNVASFGLTEQQAR, HGELLGGHLVGHDVAELLPELTLAQRWDLTASELAR
**RvD4**	Rv2953	*Trans*-acting enoyl reductase	42	6.2	20%	MMLGPNAADWPLILADASQPLTLEAMAAR, STAVLLAQSGLALALDRDR
RvD5	Rv2145c	Wag31	35	4.5	45%	INELDQELAAGGGAGVTPQATQAIPAYEPEPGK, TNTAKAESDK, TRLKTYLESQLEELGQ, GSAAPVDSNADAGGFDQFNRGK
RvD6	Rv3028c	Probable electron transfer flavo protein	32	4.5	8%	TVSPQLYIALGISGAIQHR
RvD7	Rv1886c	Ag85B mycolyl transferase	30	5.3	24%	VQFQSGGNNSPAVYLLDGLR, NDPTQQIPK
RvD8	Rv2780	Secreted L-alanine dehydrogenase ALD	32	6.1	21%	GHEVLIQAGAGEGSAITDADFK, ADLVIGAVLVPGAK, TSTYALTNATMPYVLELADHGWR
**RvD9**	Rv0054c	Single-strand binding protein Ssb	23	4.6	12%	FTPSGAAVANFTVASTPR, ASRSGGFGSGSR
RvD10	Rv0854	Conserved protein	20	4.9	6%	TAGITDEQVVAYSWTDR
**RvD11**	Rv2185c	Conserved protein TB16.3	19	5.1	18%	TTQTIYIDADPGEVMK, EVEILEADDEGYPKR, GSGTEVTYELAVDLAVPMIGMLK
**RvD12**	Rv1284	Beta-carbonic anhydrase	18	5.4	21.50%	TVTDDYLANNVDYASGFK, GFVFDVATGK
**RvD13**	Rv2031c	Heat shock protein (HSPX)	16	4.9	29.0%	ATTLPVQRHPR, AELPGVDPDKDVDIMVR, SEFAYGSFVR, HIQIR
RvD14	Rv2445c	Probable nucleoside diphosphate kinase K (NDK)	15	5.3	22.80%	GLTIAALQLR
RvD15	Rv3418c	10 kDa chaperonin GROES	12	4.4	58%	IPLDVAEGDTVIYSK, RIPLDVAEGDTVIYSK

*Details of proteins characterization by mass spectrometry from the anaerobic cultures (designated as RvD) of H37Rv. Bold underlined spot numbers indicate the newly appearing proteins during hypoxia.*

Among the 15 protein spots, 9 (RvD 1, 2, 5, 6, 7, 8, 10, 14, and 15) were over-expressed as a result of hypoxia in H37Rv and characterized as Rv0440 (GroEL2), Rv1240 [probable malate dehydrogenase (MDH)], Rv2145c (Wag31), Rv3028c (Flavoprotein), Rv1886c (Ag85B), Rv2780 (alanine dehydrogenase), Rv0854 (conserved protein), Rv2445c (diphosphate kinase), and Rv3418c (GroES), respectively. Newly appeared spots during depleted oxygen conditions in H37Rv were RvD 3, 4, 9, 11, 12, and 13 and identified as Rv0462 (dihydrolipoamide dehydrogenase), Rv2953 (enoyl reductase), Rv0054c (single strand stabilizing protein), Rv2185c (TB16.3), Rv1284 (β-carbonic anhydrase), and Rv2031c [HspX (Heat Shock Protein)], respectively.

Eleven protein spots increased in intensity (over-expressed) and six newly appeared spots were identified in S7 during hypoxia, designated as S7D (dormant S7) and sequentially numbered in the gel (**Figures 2C,D**). Their identifications are given in **Table 2**. Interestingly, Rv0440-GroEL2 (spot no. S7D1) was also identified in H37Rv (spot RvD1) under hypoxia, hence considered to be a common protein spot between S7 and H37Rv. In addition, Rv2445c was also identified as over-expressed and common to both S7 (S7D16) and H37Rv (RvD14).

**Table 2:** Details of overexpressed and newly appearing proteins identified by mass spectrometry from *M. tuberculosis* laboratory strain S7 under hypoxia compared to aerated cultures.

Spot no	Rv no	Protein Name	Predicted M.Wt (kDa)	Predicted pI	Coverage (%)	Peptide matched
S7D1	Rv0440	GroEl2	65	4.8	11%	GLNALADAVK, EIELEDPYEK
**S7D2**	Rv3667	Acetyl coA synthetase	80	5.9	10%	LLITSDGQFRR, RGHPAPLKDAADEAVSQPDSPVEHVLVVRRTGIDVSWNDER, AEVAEAISPIARPR
S7D3	Rv0405	Polyketide synthase-6 (pks6)	110	5.4	1%	TASALAAQAGRLGR
**S7D4**	Rv2220	Glutamine synthetase	62	5.3	16.70%	SVFDDGLAFDGSSIR, GGYFPVAPNDQYVDLR, MLTNINSGFILEK, LVPGYEAPINLVYSQR
**S7D5**	Rv1679	Possible acyl-CoA dehydrogenase FadE16	55	4.9	23%	VDTDCAFPAEAVDALRK, ASVNDAALTITESAMR
**S7D6**	Rv2953	*Trans*-acting enoyl reductase	42	6.1	20%	MMLGPNAADWPLILADASQPLTLEAMAAR, STAVLLAQSGLALALDRDR
**S7D7**	Rv3029c	Probable electron transfer flavo protein beta subunit	31	4.8	4.90%	EAADAVLDEINER
S7D8	Rv3060c	GntR family transcriptional regulator	33	5.2	2.30%	TYGASGMPSR
S7D9	Rv0632c	Probable enoyl-CoA hydratase EchA3	25	5.8	13.80%	VFSGGFDLK, GGFELAYR, ILTSGEVQPAIDMLR
S7D10	gi| 183985444	Integral membrane protein	21	4.7	0.90%	GPIPFDAPRER
**S7D11**	gi| 518086696	Cyclase [*M. tuberculosis*]	20	4.8	11.10%	AIADIEAYPQWISEYK
S7D12	Rv3716c	Conserved protein	15	4.4	19%	VVDPDDIETLQDLIVGAMR
S7D13 & 14	Rv2031c	Heat shock protein (HspX)	16	4.6	29%	ATTLPVQRHPR, AELPGVDPDKDVDIMVR, SEFAYGSFVR
S7D15	Rv3418c	10 kDa chaperonin (GROES)	12	4.2	58%	IPLDVAEGDTVIYSK, RIPLDVAEGDTVIYSK
S7D16	Rv2445c	Probable nucleoside diphosphate kinase K (NDK)	15	5.3	22.80%	GLTIAALQLR
S7D17	Rv3875	ESAT-6	12	4.3	17.90%	WDATATELNNALQNLAR

*Details of proteins characterization by mass spectrometry from the S7anaerobic cultures (designated as S7D). Bold underlined spot numbers indicate the newly appearing proteins during hypoxia. For few spots gene number was not described in protein search tool, in such case accession number was given.*

Rv2953, *trans*-acting enoyl reductase, appeared newly in both S7 (S7D6) and H37Rv (RvD4) during hypoxia. Rv2031c which was identified as a newly appeared spot in H37Rv (RvD13) was identified as overexpressed and present as multiple spots in S7 during hypoxia (S7D13 and S7D14).

Protein expression in the clinical strain S10, under aerobic and anaerobic conditions is given in **Figures 2E,F**. Hypoxia induced protein spots in S10 were designated as S10D (dormant S10), numbered sequentially and spot characterization by mass spectrometry is given in **Table 3**. As observed in H37Rv and S7, Rv0440-GroEL2 was also found to be overexpressed under hypoxia in S10 (S10D1). Hence, it is grouped with “common spots.”

**Table 3:** Details of overexpressed and newly appearing proteins identified by mass spectrometry from *M. tuberculosis* laboratory strain S10 under hypoxia compared to aerated cultures.

Spot no	Rv no	Protein Name	Predicted M.Wt (kDa)	Predicted pI	Coverage (%)	Peptide matched
S10D1	Rv0440	GroEl2	65	4.8	11%	TDDVAGDGTTTATVLAQALVR
S10D2	Rv0350	Probable chaperone protein DNAK	75	4.9	23%	HMGSDWSIEIDGKK, GIPQIEVTFDIDANGIVHVTAK
S10D3	Rv0462	Dihydrolipoamide dehydrogenase (LpdC)	50	5.8	25%	VAGVHFLMK, ATFCQPNVASFGLTEQQAR
S10D4	Rv1240	Probable malate dehydrogenase (MDH)	48	4.9	16.7%	NAAEVVNDQAWIEDEFIPTVAK, SDLLEANGAIFTAQGK
S10D5	Rv0351	GrpE	27	4.2	31%	PDGNSGEQVTVTDKRR, PVIGTVMRQGYQLGEQVLR
S10D6	Rv3029c	Probable electron transfer flavo protein (β-subunit)	31	4.8	4.90%	EAADAVLDEINER
S10D7	Rv2032	Conserved protein acg	30	5.7	7%	IDVIADDMRPELAAASK
S10D8	gi| 33742142	Sequence 142 from patent US 6583266 (corresponding atpA)	23	4.2	3.70%	VVNPLGQPIDGR, GTTIASVRR
**S10D9**	Rv3841	Possible bacterioferritin (BfrB)	20	4.3	13.30%	VEIPGVDTVR, AGANLFELENFVAR
S10D10	Rv3841	Possible bacterioferritin (BfrB)	20	4.6	42%	NHAMMLVQHLLDR, VEIPGVDTVR, EALALALDQER, VALMATLVR, AGANLFELENFVAR, EVDVAPAASGAPHAAGGRL
S10D11	gi| 518086696	Cyclase [*M. tuberculosis*]	20	4.8	20.80%	AIADIEAYPQWISEYK, QSLSWTLESSSLLK
S10D12	Rv3716c	Conserved protein	15	4.4	19%	VVDPDDIETLQDLIVGAMR
S10D13	Rv2031c	HspX	16	4.7	29%	ATTLPVQRHPR, AELPGVDPDKDVDIMVR, SEFAYGSFVR
S10D14	Rv1636	Iron-regulated universal stress protein family protein TB15.3	14	5.8	37%	LIIASAYLPQHEDAR, DESYKVTGTAPIYEILHDAK, LLGSVPANVSRRAK
S10D15	Rv3418c	10 kDa chaperonin (GROES)	12	4.5	15%	IPLDVAEGDTVIYSK
S10D16	Rv3418c	10 kDa chaperonin GROES	12	4.5	27%	IPLDVAEGDTVIYSK, YNGEEYLILSAR
S10D17	Rv2626c	Hypoxic response protein 1 (Hrp1)	12	4.7	9%	DIMNAGVTCVGEHETLTAAAQYMR

*Mass spectrometry characterization of anaerobic proteins of S10 (designated as S10D). Underlined spot numbers indicate the newly appearing proteins during hypoxia. For few protein spots accession number was given since the gene number was not described in protein search tool used here.*

**FIGURE 3:**
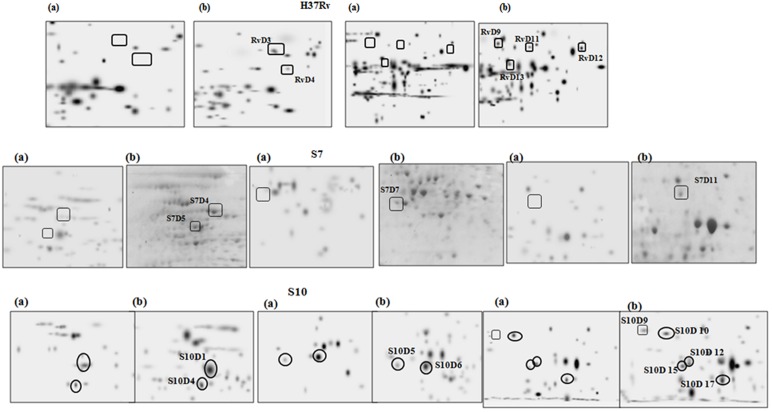
**Magnified regions of gel pictures showing proteins (a) aerobic cultures (b) anaerobic cultures from H37Rv, S7, and S10**.

A well known hypoxia protein α-crystalline, encoded by Rv2031c, was also found to be overexpressed in S10 (S10D13) and found to be a “common spots” in all three strains of the present study. Apart from this, the protein spot Rv0462 (dihydrolipoamide dehydrogenase) and Rv1240 (MDH) were identified as common spots between H37Rv and S10. Magnified regions of gel portions are given in **Figure 3** for better visualization and representative MS/MS data for a randomly selected spot from each strain is given in Supplementary Figure S1.

### Functional Classification of Differentially Expressed Proteins

The majority of differentially expressed proteins from H37Rv were predicted to be involved in “Intermediary metabolism and respiration” (Rv0462, Rv1240, Rv1284, Rv2445c, and Rv3028c). In case of S7, the majority of proteins were classified under “lipid metabolism” (Rv0405, Rv0632c, Rv1679, Rv2953, and Rv3667) and in S10, expressed proteins were predicted to be involved in virulence, detoxification, and adaptation (Rv0350, Rv0351, Rv0440, Rv1636, Rv2031c, and Rv3418c).

Proteins involved in cell wall synthesis (Rv2145c, Rv3875) and conserved hypothetical proteins were also found to be differentially expressed during hypoxia and their distributions among the strains are given in **Table 4**.

**Table 4:** Categorization of over expressed and newly appeared proteins under hypoxia in H37Rv, S7, and S10.

S. No	Classifications	H37Rv	S7	S10
1	Amino acid biosynthesis	None	Rv2220	None
2	Cell envelope	Rv0440, Rv2145c	Rv0440	Rv0440
3	Cellular processes	None	Rv0405, Rv3875	Rv1636
4	Central intermediary metabolism	Rv1284	None	None
5	Energy metabolism	Rv0462, Rv2780, Rv1240	Rv1679	Rv0462, Rv1240
6	Hypothetical proteins–Conserved	Rv0854, Rv2185c	Rv3716c	Rv2032
7	Protein fate	None	Rv3418c, Rv3875	Rv3418c
8	Regulatory functions	None	Rv3060c	None
9	Transport and binding proteins	None	None	Rv3841
10	Unclassified	Rv1886c, Rv2031c, Rv2445c, Rv2953, Rv3028c	Rv0632c, Rv2031c, Rv2445c, Rv2953, Rv3029c, Rv3667	Rv0350, Rv0351, Rv2031c, Rv2626c, Rv3029c

*Over-expressed and newly appeared proteins from H37Rv, S7 and S10 during hypoxia was categorized according to their predicted biological functions based on the information given in TubercuList database.*

### Higher Frequency of Memory T Cell Markers in Circulation of Healthy Infected Individuals

**FIGURE 4:**
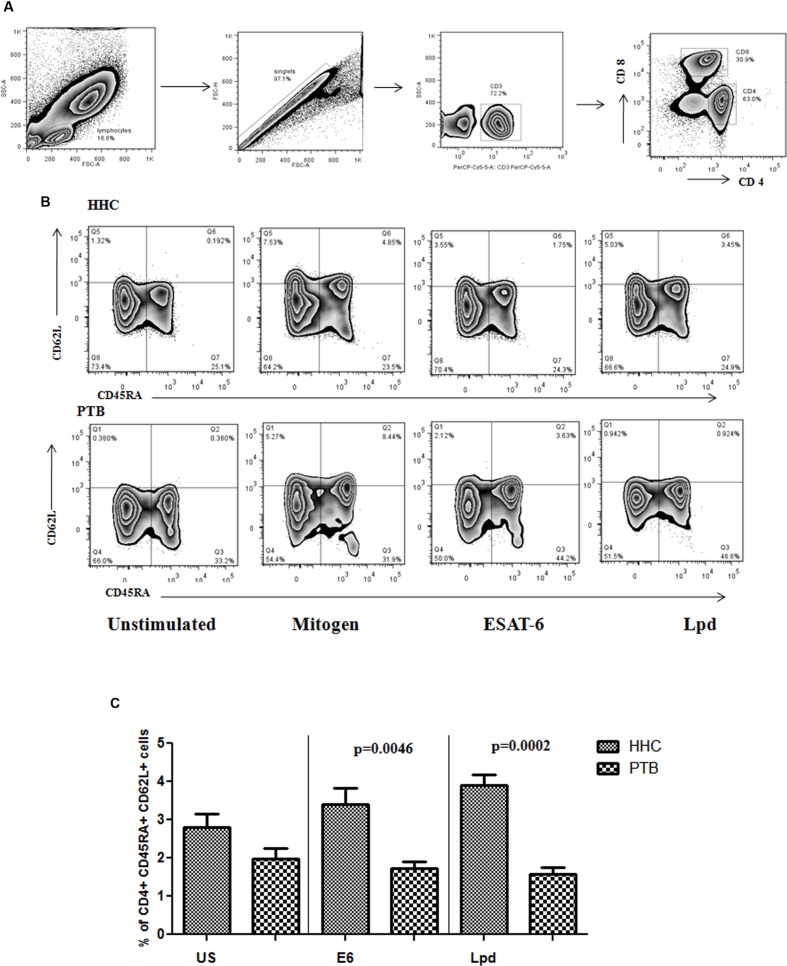
**(A)** General gating strategy for T cell is given. After selecting the singlets from lymphocyte population, CD3^+^ cells were gated followed by CD4^+^ cells were gated and selected for memory phenotyping. **(B)** CD4^+^ cells were analyzed for the expression of CD62L and CD45RA surface markers to define the subtypes of memory cells. Antigen specific expansion of the central memory phenotype is evident in healthy household contacts (HHC) and representative flow diagram from HHC and pulmonary TB (PTB) is given. **(C)** Percentage of central memory cells specific to ESAT-6 (E6) and Lpd were shown as bar graphs representing mean with SEM. *p*-values were calculated using the Mann–Whitney *U* test and value <0.05 considered as significant.

Antigen specific memory T cells were analyzed in the stimulated blood culture of HHC and PTB. The gating strategy followed (**Figure 4A**) along with representative flow cytometry is given in **Figure 4B**. Significantly higher antigen specific memory cells were present in HHC, ESAT-6 (*p* < 0.005), and for Lpd (*p* < 0.0005) **Figure 4C**, when compared to PTB with respect to central memory cell phenotype. Mitogen response, shown only in the representative flow diagram, was equal in both HHC and PTB showing proliferative capacity was not defective. These antigens specific Th1, Th2, and poly functional T cell response was also found to be significantly higher in HHC (*N* = 30) when compared to PTB (*N* = 30; Communicated manuscript).

## Discussion

Laboratory strains (such as H37Rv) might not completely mimic the virulence of naturally occurring clinical strains. Vaccines based on proteins that are predicted to be over-expressed only in the laboratory strain might not prevent infections caused by virulent strains. Hence, we have included the most prevalent clinical *M. tuberculosis* strains (S7 and S10) to study protein expression under hypoxia. In our experiments, growth related differences were minimized between the strains by terminating all cultures during late exponential growth (25–30 days) and expression of already reported hypoxia genes and DosS–DosR regulon genes shows faithful achievement of hypoxia (Devasundaram et al., 2015). For a few protein spots, the percentage of peptide coverage was less than 10, which is still acceptable (Mehaffy et al., 2010; Ang et al., 2014) and could be due to the lowest concentration of peptides obtained from the protein spots.

The expression of molecular chaperones (assist proper protein folding) and proteases (degrades unfolded proteins) was observed to be an adaptive bacterial response during various environmental stresses (Gumber and Whittington, 2009). DosR antigens and members of the small heat-shock protein family, also known as chaperones, were identified as over-expressed during hypoxia in all three strains H37Rv, S7, and S10. The small heat shock protein family includes chaperones like Rv2031c [α-crystallin (ACR) protein–HspX], Rv3418c (10 kDa chaperonin GroES), and Rv0440 (Hsp65), encoded in the second copy of a gene for a 60 kDa chaperonin in the *M. tuberculosis* genome and all are identified in the present study.

GroEL2 (Rv0440), also a chaperone, prevents protein misfolding, and promotes the refolding and proper assembly of unfolded/misfolded polypeptides generated during stress conditions (Sharma et al., 2010). This protein was predicted to be over-expressed in all three strains under hypoxia. GroES (Rv3418c), also a chaperone protein, was already reported to be expressed by Rustad et al. (2009) and our results also confirmed overexpression during hypoxia. Collectively, this shows hypoxia associated proteins’ expression in our experimental model.

A number of reports observed the expression of HspX (Rv2031c) under hypoxia (Starck et al., 2004; Siddiqui et al., 2011), and its expression was also observed during aerobic growth based on H37Rv as a model strain (Desjardin et al., 2001). Adding to these reports, we also observed no change in expression of HspX in H37Rv under hypoxia. Since HspX expression was not unique to hypoxia, as observed based on H37Rv protein analysis, many researchers might exclude to target them for vaccine development. But, in contrast to H37Rv, HspX was identified as over-expressed in both the clinical isolates (S7 and S10) during hypoxia indicating its possible role during hypoxia in clinical isolates. This further highlights the need to include the most relevant clinical strains for *in vitro* experiments to minimize bias.

Heat shock protein accumulates as the dominant marker during LTBI, which is estimated to affect almost one-third of the world’s population (Dubaniewicz et al., 2013). Hence, HspX can still be considered a hypoxia associated protein. In our experiments, an additional new spot of HspX was found adjacent to the actual HspX spot position which might have been a result of proteolytic degradation or post-translational modification. Different spots representing HspX (isoforms or degraded spots) at lower molecular weights with differing pI have already been observed during 2-DE analysis of mycobacterial proteins (Betts et al., 2000).

Proteins of the Acr family were reported to be involved in the oxidative stress response in *M. tuberculosis* and persistence mechanisms in host macrophages (Stewart et al., 2005). Its expression during heat shock stress response was also observed (Gumber and Whittington, 2009); but its role under hypoxia in clinical isolates (S7 and S10) is evidently reported by our results. This observation extends support for the ACR family proteins to still serve as a better target for understanding the latency mechanisms of *M. tuberculosis*.

The lipid-rich outer cell wall layer, a unique feature of mycobacteria, contributes to their resilience and contains many compounds known to be involved in virulence (Camacho et al., 2001). Phthiocerol dimycocerosate (PDIM) constitutes a major virulence factor and functionally important surface-exposed lipid of *M. tuberculosis.* Biosynthesis of the PDIM core domain requires gene *ppsD* that encodes one of the five modular type-I polyketide synthases (*ppsA–E*) of *M. tuberculosis* (Perez et al., 2004). But *ppsD* lacks functional enoyl reductase activity which is required for the synthesis of these lipids. Rv2953 encodes a trans-acting enoyl reductase that acts along with *ppsD* in phthiocerol and phenolphthiocerol biosynthesis and completes the final steps in PDIM biosynthesis (Simeone et al., 2007). We observed over-expression of Rv2953 in the most prevalent clinical isolates and H37Rv in our hypoxia experiment. This supports the idea that Rv2953 expression is needed during dormancy, where thickening of mycobacterial cell walls is generally observed, to complete cell wall lipid biosynthesis (PDIM; Murry et al., 2009). This strengthens the idea of targeting Rv2953 for antibody development to neutralize virulence factor biosynthesis or for use as a biomarker for latency.

The second common overexpressed protein between H37Rv and S7 was Rv2445c (nucleoside phosphate kinase-Ndk). Ndk is known for its interactions with host signaling molecules Rab5 and Rab7 (Hop et al., 2015), two small GTPases that control phagosome lysosome fusion, the consequence of which is inhibition of phagolysosome fusion (Dar et al., 2011). Ndk can also interact with the host Rac1 signaling molecule that leads to an NADPH oxidase assembly; defect in this assembly would cause impaired reactive oxygen species production (Sun J. et al., 2010). In doing so, Ndk contributes to intracellular survival and subsequent establishment of mycobacterial infection. Though the GTPase activity of Ndk has been reported earlier (Chopra et al., 2003) the contribution of Ndk to *M. tuberculosis* pathogenesis has only recently been addressed (Sun J. et al., 2010; Sun J.N. et al., 2010). Ndk expression was not observed in any of the earlier *in vitro* based *M. tuberculosis* stress model studies. A recent nutrient starvation model study revealed Ndk expression, but with no significant difference in protein expression (Albrethsen et al., 2013). Though the role of Ndk in *M. tuberculosis* was well elucidated, its role during hypoxia has not been clarified so far. We report for the first time, to our knowledge, on the possible role of Ndk during hypoxia. We identified Rv1240 (MDH) and Rv0462 (dihydrolipoamide dehydrogenase) as over expressed proteins common between H37Rv and S10. It is a well-known fact that during the persistent phase of infection, *M. tuberculosis* switches to tricarboxylic acid (TCA) metabolism to utilize fatty acids as a carbon source (Bishai, 2000). Thus, TCA metabolic enzymes are likely to have a role during mycobacterial dormancy. MDH (Rv1240), a TCA metabolic enzyme, was found to be unique to intraphagosomal mycobacteria (Mattow et al., 2006), which supports its overexpression under hypoxia, a condition observed within phagosomes. Along with these observations, our results also highlighted the possible role of Rv1240 during hypoxia.

Lpd (Rv0462), *M. tuberculosis’*s sole dihydrolipoamide dehydrogenase (Argyrou and Blanchard, 2001) is a flavin-adenine dinucleotide-containing NADH-dependent oxidoreductase that plays an essential role in intermediary metabolism as the E3 component of the pyruvate dehydrogenase complex. Earlier reports indicated that *M. tuberculosis* becomes vulnerable when Lpd (Rv0462) is inhibited (Bryk et al., 2002) and Lpd helps *M. tuberculosis* to resist host reactive nitrogen intermediates (Venugopal et al., 2011). These dynamics clearly convey a role for the metabolic enzymes Rv1240 and Rv0462 during hypoxia. *Lpd* gene expression under hypoxia was not reported earlier and a unique feature of our results are that they show the first evidence for Lpd expression during hypoxia as well as its expression in clinical strains of *M. tuberculosis*.

The electron transfer flavoprotein β subunit (Rv3029c) and a conserved hypothetical protein (Rv3716c) were found exclusively in both the clinical isolates S7 and S10 as over-expressed proteins during hypoxia, but not in H37Rv. Rv3029c is a well-known gene that participates in β-oxidation of fatty acids (Covert et al., 2001) and produces energy when fatty acids are used as the sole carbon source during persistence. Not much data is available for Rv3716c, but it was reported under “contact specific antigens” in a study conducted on healthy contacts of TB and PTB patients using H37Rv culture filtrate antigen fractions (Deenadayalan et al., 2010).

Rv3667 (acetyl CoA synthetase) and Rv2220 (glutamine synthetase), well known genes of hypoxia from H37Rv reported by many studies (Sherman et al., 2001; Rustad et al., 2009) were found only in the S7 strain, in addition to Rv3875 (ESAT-6) and Rv1679 (FadE6). In contrast, gene Rv0405 (polyketide synthase-6) reported as repressed under hypoxia with H37Rv (Rustad et al., 2009) was found to be expressed during hypoxia in the clinically prevalent strain S7. Genes Rv1679 (possible FadE16), Rv3060c (GntR family transcription regulator), Rv0632c (probable enoyl-coA hydratase) were not found to be reported from H37Rv-based *in vitro* hypoxic model, but appeared in the S7 clinical strain. We were tempted to speculate on differences between the laboratory and clinically prevalent strains, given similar treatment conditions and to create a list of possible true representative antigens. But, these variations were found only with S7 and not with S10, which responded similarly to H37Rv.

Our earlier microarray study with total RNA from these three strains under aerobic and anaerobic conditions also found similar hypoxic gene expression patterns during hypoxia between S10 and H37Rv. Whereas gene expression in S7 differed from the laboratory strain H37Rv (Devasundaram et al., 2015). Thus, studying the most prevalent virulent strains would help to better understand their pathogenesis and minimize variations in targeting virulence factors for effective control of infectious diseases.

The complete list of genes that are predicted to be overexpressed and common in all three strains during hypoxia was discussed in our earlier microarray report. In contrast, the present mass spectrometry report is preliminary and total spot characterization has yet to be completed. Hence, the 134 common hits identified during microarray reporting might not correlate completely with our present mass spectrometry data. But, a few characterized protein spots like Rv2953 (RvD4), Rv3060c (S7D8), Rv1240 (S10D4) was also found in our gene expression data (GEO accession no: GSE55863).

Cellular immune response in LTBI would reflect the type of immunity responsible for efficient disease control and serve as a good experimental model (Kumar et al., 2010). The availability of two antigens in our lab, ESAT-6 and Lpd allowed us to evaluate the predicted antigens from the established *in vitro* oxygen depletion model in human experimental set-up. Similar outcomes were observed with both peripheral blood mononuclear cells (PBMC) and whole blood (WB) during T lymphocyte assays. WB assays are advantageous compared to PBMC assays since they require less blood volume. Hence, we preferred to stimulate WB to assess T-lymphocyte response. Generally many researchers dilute the blood with RPMI to screen more antigens simultaneously at dilutions of 1:1 (Kumar N.P. et al., 2013), 1:2 (Kumar et al., 2010), 1:5 and 1:10 (Deenadayalan et al., 2013) and we followed 1:2 as was followed by (Kumar et al., 2010). Antigen specific central memory cells were identified in HHC showing their association during latency. It has already been shown that CD4^+^ memory T cells are predominant functional subsets in LTBI (Pollock et al., 2013). The decreased frequency of central memory cells in PTB shows the absence or decline of antigen (ESAT-6, Lpd) specific memory cells in the circulation of active TB patients. This clearly suggests that these antigens are expressed in the dormancy state and have a possible role during latency. Since latent TB populations are considered to be “protective against active TB disease,” antigens that are predominantly recognized by their sensitized T cells could be a potential target for vaccine development. Global searches have allowed the identification of more than a 100 potential virulence genes in pathogenic mycobacteria. But, controlling TB infection is still a major challenge due to the distinct behavior of the laboratory model strains and the clinical strains. Thus, it is crucial to study clinically relevant infectious strains to identify common, abundant proteins between strains for drug development and vaccines. Putative drug targets, vaccine candidates, and diagnostic markers for TB were identified by comparative proteome analyses of *M. tuberculosis* strains/clinical isolates of varying virulence. Thus genes/proteins observed from our results can be explored further for their use in diagnosis or vaccine development against TB.

## Author Contributions

Conceived and designed the experiments: AR and SD. All the experiments and analysis was performed: SD. Mycobacterial culture work and 2-DE work assisted: AG. All authors contributed equally for manuscript writing.

## Conflict of Interest Statement

The authors declare that the research was conducted in the absence of any commercial or financial relationships that could be construed as a potential conflict of interest.

## Footnotes

1 http://www.ncbi.nlm.nih.gov/refseq/

2 http://tuberculist.epfl.ch/
