# Supplementary material for: Proteomics Analysis of Three Different Strains of Mycobacterium tuberculosis under In vitro Hypoxia and Evaluation of Hypoxia Associated Antigen’s Specific Memory T Cells in Healthy Household Contacts
Source: Front Microbiol. 2016 Sep 9;7:1275. doi: 10.3389/fmicb.2016.01275 (PMC5017210; doi:10.3389/fmicb.2016.01275)
Supplement: Supplementary file 1 [file Data_Sheet_1.DOC]

**Proteomics analysis of 3 different strains of *Mycobacterium tuberculosis* under *in vitro* hypoxia and evaluation of hypoxia associated antigen’s specific memory T cells in healthy household contacts**

**AUTHORS:**

**Santhi Devasundaram**, Akilandeswari Gopalan Sulochana D Das and Alamelu Raja*

**ADDRESS:**

Department of Immunology,

National Institute for Research in Tuberculosis (ICMR),

(Formerly Tuberculosis Research Centre)

No.1, Mayor Sathyamoorthy Road, Chetpet, Chennai - 600 031. India.

***CORRESPONDING AUTHOR AND REPRINT REQUEST:**

Dr. Alamelu Raja,

National Institute for Research in Tuberculosis (ICMR),

(Formerly Tuberculosis Research Centre)

No.1, Sathyamoorthy Road, Chetpet,

CHENNAI - 600 031, INDIA.

E-mail: [alameluraja@gmail.com](mailto:alameluraja@gmail.com)

Phone: +91 (044) 2836 9682

FAX: +91 (044) 2836 2528

**Fig.S1. Representative mass spectrometry data of random spots from each strain**


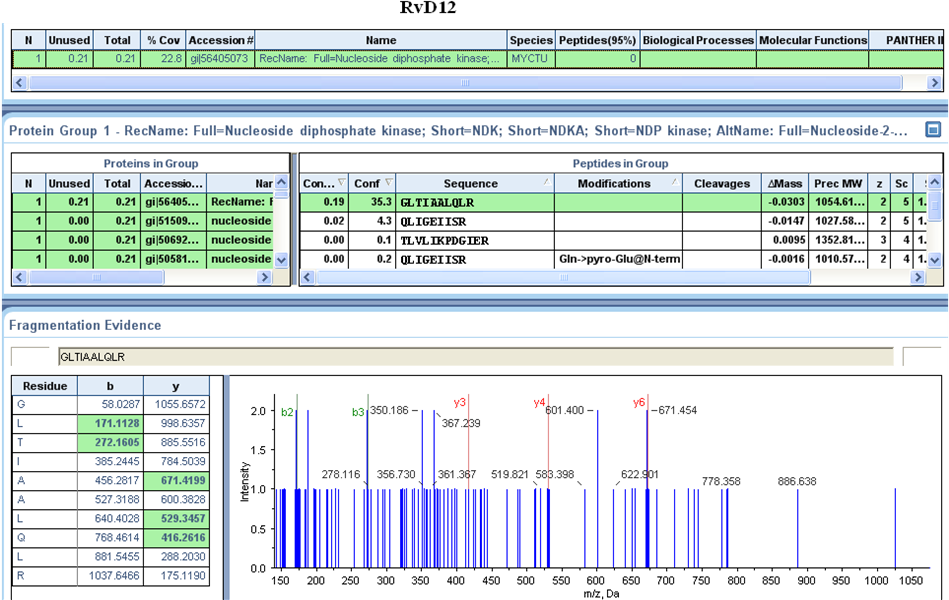


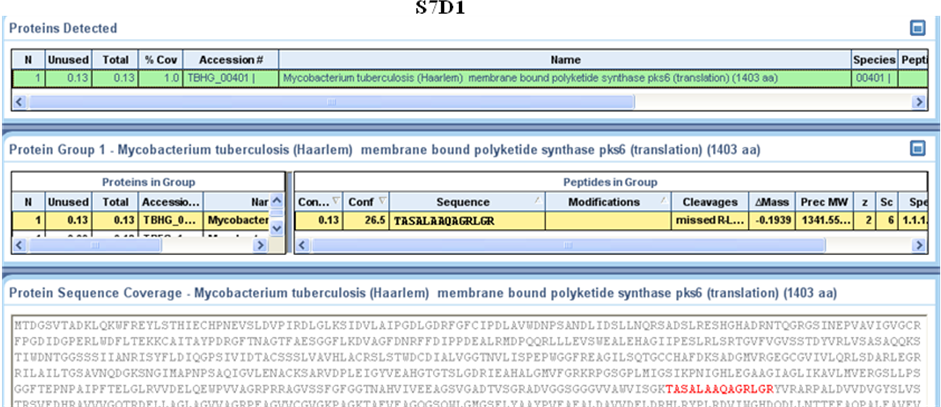


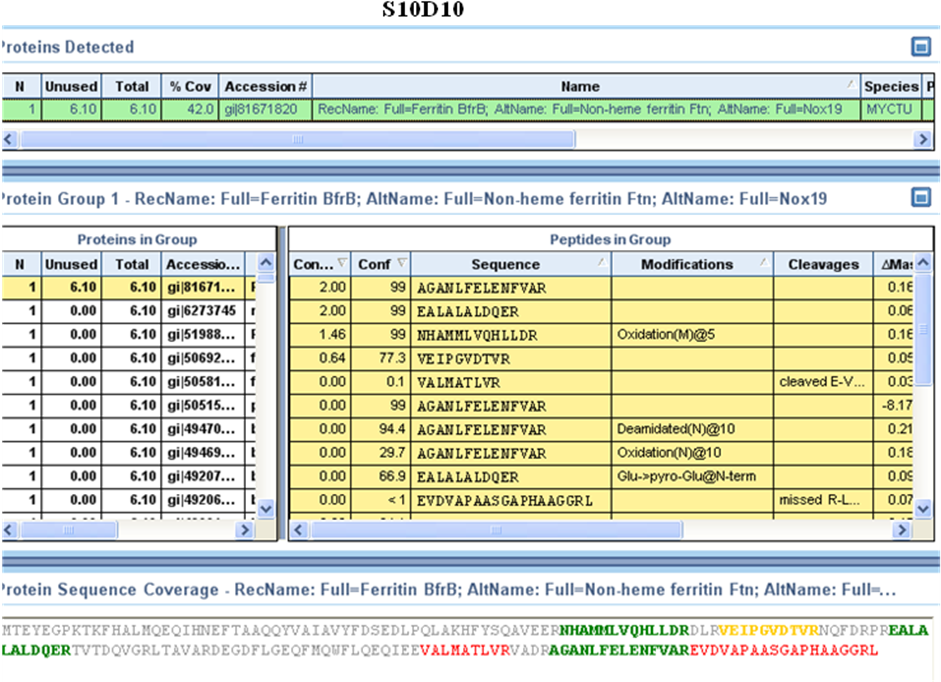


Representative images of results of peptide search using Proteinpilot software is given for each strain.
